# Supplementary material for: Causal Relationship between Aging and Anorexia Nervosa: A White-Matter-Microstructure-Mediated Mendelian Randomization Analysis
Source: Biomedicines. 2024 Aug 16;12(8):1874. doi: 10.3390/biomedicines12081874 (PMC11351342; doi:10.3390/biomedicines12081874)
Supplement: Supplementary file 1 [file biomedicines-12-01874-s001.zip › Supplementary Figure/Figure S7/Single SNP plot of MR analysis for ubm-b-1916 to AN.pdf]

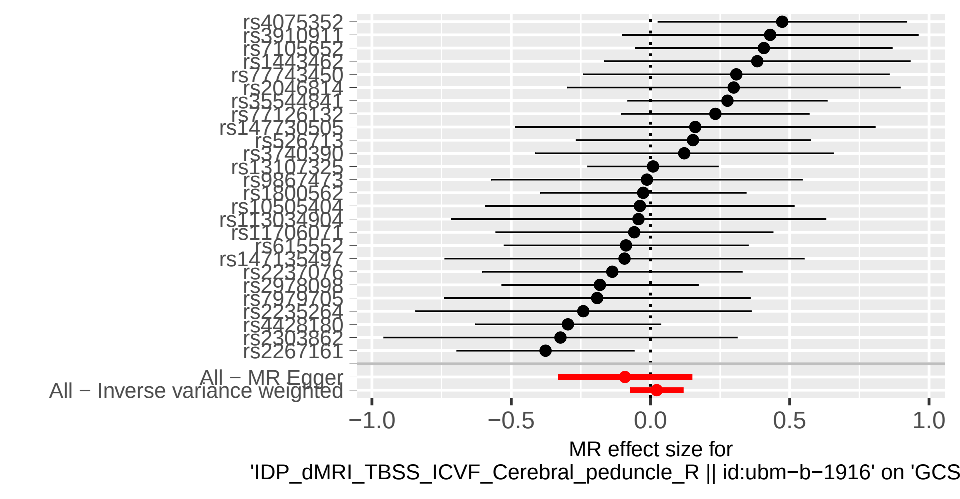
rs4075352
rs3910911
rs7105652
rs1443462
rs77743450
rs2046814
rs35544841
rs77126132
rs147730505
rs526713
rs3740390
rs13107325
rs9867473
rs1800562
rs10505404
rs113034904
rs11706071
rs615552
rs147135497
rs2237076
rs2978098
rs7979705
rs2235264
rs4428180
rs2303862
rs2267161
All − MR Egger
All − Inverse variance weighted
−1.0
−0.5
0.0
0.5
1.0
MR effect size for
'IDP_dMRI_TBSS_ICVF_Cerebral_peduncle_R || id:ubm−b−1916' on 'GCS
